# Tomosyn Interacts with the SUMO E3 Ligase PIASγ

**DOI:** 10.1371/journal.pone.0091697

**Published:** 2014-03-10

**Authors:** Cornelia J. Geerts, Linda Jacobsen, Rhea van de Bospoort, Matthijs Verhage, Alexander J. A. Groffen

**Affiliations:** 1 Department of Functional Genomics, Center for Neurogenomics and Cognitive Research, Neuroscience Campus Amsterdam, VU University, Amsterdam, Netherlands; 2 Department of Clinical Genetics, VU Medical Center, Amsterdam, Netherlands; University of Toronto, Canada

## Abstract

Protein modification by Small Ubiquitin-like MOdifier (SUMO) entities is involved in a number of neuronal functions, including synaptogenesis and synaptic plasticity. Tomosyn-1 (syntaxin-binding protein 5; STXPB5) binds to t-SNARE (Soluble NSF Attachment Protein Receptor) proteins to regulate neurotransmission and is one of the few neuronal SUMO substrate proteins identified. Here we used yeast two-hybrid screening to show that tomosyn-1 interacts with the SUMO E3 ligase PIASγ (Protein Inhibitor of Activated STAT; PIAS4 or ZMIZ6). This novel interaction involved the C-terminus of tomosyn-1 and the N-terminus of PIASγ. It was confirmed by two-way immunoprecipitation experiments using the full-length proteins expressed in HEK293T cells. Tomosyn-1 was preferentially modified by the SUMO-2/3 isoform. PIASγ-dependent modification of tomosyn-1 with SUMO-2/3 presents a novel mechanism to adapt secretory strength to the dynamic synaptic environment.

## Introduction

SUMOylation involves covalent attachment of a 12 kDa Small Ubiquitin-related MOdifier (SUMO) protein to a protein substrate. In mice at least 3 functional SUMO isoforms exist, of which SUMO-2 and -3 are highly similar. The mechanisms behind SUMO-isoform selectivity remain elusive, as well as their functional differences [1]. SUMO has been studied mainly as a regulator of gene expression by SUMOylation of transcription factors [2], [3] and histones [4] and as a modulator of nuclear localization [5], [6], but is also involved in cell cycle regulation and DNA repair [7]. Protein SUMOylation can lead to altered protein interactions via SUMO interacting motifs [8]. In neurons, SUMOylation modulates spine development, spine structure and function as well as synaptogenesis and synaptic plasticity [9]. The latter could be mediated by activity-dependent redistribution of the SUMOylation machinery [10]. SUMO-2/3 attachment is increased during ischemic stress in neurons, which could serve as a neuroprotective mechanism [11], [12], [13], [14], [15]. Additionally, SUMOylation has been indicated to play a role in several neurodegenerative diseases [16], [17]. Nevertheless, only a few neuronal SUMO substrates have been identified (Reviewed in [17]).

Similar to ubiquitin modification, SUMOylation is directed by the subsequent actions of E1, E2 and E3 ligases [1]. SUMO E2 ligase Ubc9 deficient mice die at early embryonic stages, emphasizing the importance of the SUMOylation pathway [18]. Although E3 ligases are not essential for protein SUMOylation, they may particularly enhance efficiency and selectivity of SUMO attachment to non-consensus motifs. PIAS (Protein Inhibitor of Activated STAT) family proteins are known SUMO E3 ligases, interacting directly with Ubc9 [3] and initially identified as inhibitors of the JAK-STAT signalling pathway [19]. The family consists of PIAS1, PIAS3, PIASxα, PIASxβ, and PIASγ. PIASγ (or PIAS4/ZMIZ6) is, with 507 amino acids, the shortest PIAS family member and localizes primarily to the nucleus [2]. The RING finger domain of PIASγ is indispensable for its E3 ligase activity [20], but may also execute ligase independent functions [21]. In *in situ* hybridization experiments in developing mouse embryos, PIASγ localizes to tissues of ectoderm origin, including cells in the nervous system, limb and epidermis, suggesting it is involved in the differentiation of these cells [22]. PIASγ deficient mice do not show obvious abnormalities [23], perhaps because of compensatory mechanisms. PIAS1/PIASγ double knock-out mice die prematurely [24], indeed implying functional overlap between these proteins. (For a more elaborate review on PIAS proteins, see [25]). Thus, the E3 ligase PIASγ could be an important modulator of SUMO attachment in neurons.

Tomosyn is an inhibitor of vesicular secretion that has been studied in the context of synaptic transmission [26], [27], [28], [29], [30] and neurite outgrowth [31], [32], as well as vesicle fusion in (neuro)endocrine cells [33], [34], [35], [36], [37], [38]. In the mouse nervous system, two tomosyn genes are transcribed, in total expressing seven isoforms of the protein, generated by alternative splicing of the mRNA [39]. The medium sized splice variant of tomosyn-1 is subject to SUMO modification [40]. Protein kinase A (PKA)-induced phosphorylation of tomosyn at a residue near the predicted SUMOylation site contributes to synaptic plasticity [41]. Importantly, phosphorylation and SUMOylation have been suggested to co-regulate protein function [42], [43], [44]. Although both tomosyn modifications could work in concert and contribute importantly to synaptic plasticity, the mechanism governing tomosyn SUMOylation is not yet identified.

In this study we report that SUMO-2/3 modification of tomosyn-1 is mediated by its interaction with the SUMO E3 ligase PIASγ, thus identifying a novel signaling pathway of potential importance for the regulation of synaptic transmission.

## Materials and Methods

### Constructs

Bait cDNA fragments were cloned into the yeast two-hybrid bait vector pBD-Gal4. pBD-Gal4-Cter encoded amino acids 540-1116 of mouse tomosyn-m1 (Genbank accession number NP_001074813.2), while pBD-Gal4-CoiledCoil encoded amino acids 1028–1116. For immunoprecipitation experiments, a mycHis tag was cloned at the C-terminus of mouse tomosyn-m1. The construct was cloned into a pCDNA3.1 backbone vector and expressed by a CMV promoter. Full-length FLAG-human PIASγ was expressed from a construct obtained from Addgene (Plasmid number 15208; [45]).

### Yeast two-hybrid screens

A mouse brain Matchmaker cDNA library (Clontech #ML4008AH) was used in yeast two-hybrid screening, derived from BALB/c cDNA from 9–12 week-old males in the prey vector pAct2. The host strain was AH109, offering tight regulation of the HIS3 and ADE2 reporter genes by GAL4 [46]. Yeast Extract Peptone Dextrose (YPD) medium contained 10 g/L yeast extract, 20 g/L bactopeptone, 2% glucose and 0.1 g/L adenine hemisulphate (AppliChem), pH 6.5. Selective growth of yeast colonies utilized synthetic complete (SC) media lacking leucine and tryptophan. These media contained 6.7 g/L Yeast Nitrogen Base (w/o amino acids), pH 5.8, 0.62 g/L Complete Supplement Mix (w/o histidine, leucine, tryptophane and adenine; BIO 101 Systems), 2% glucose, 0.4 µM histidine (optional) and 0.1 g/L adenine hemisulphate (Sigma; optional). For plating, 20 g/L Bacto-agar (Becton, Dickinson and Company) was added to the medium.

Transformation of yeast was performed using the LiAc/ss carrier DNA/PEG method [47]. A transformation mixture was prepared from 33% w/v PEG 3350, 100 mM LiAc and 280 µg/ml boiled salmon sperm (ss) carrier DNA. This was added to 100 µl yeast cell suspension (with an OD600 between 0.6 and 0.8, washed and pre-incubated with 0.1 M sterile LiAc for 5 minutes at 30°C) and 1 µg plasmid DNA (both prey and bait). After heat shock in a water bath at 42°C for 30–45 min, cells were incubated for 3 days at 30°C on plates containing 100 mM histidine, but lacking leucine and thryptophan to select for the prey and bait plasmid respectively. Colonies were replated on histidine containing plates, plates lacking histidine alone or lacking both histidine and adenine and incubated for 2 days at 30°C. Empty bait (pBD-Gal4) and prey (pAct2) vectors were used as negative controls. Clones were identified by sequencing with Matchmaker (Clontech), CMV promoter primer (5′-GGTAGGCGTGTACGGTGGGAGGTC-3′) and PIASγ specific primers (5′-GCTGTATGAGACTCGCTATGC-3′, 5′- CTCACCTTCCTCCTGCCTTAG-3′, 5′-TGCTTCAGCCCACTCTTGC-3′) and performing a BLAST search. Prey clones were confirmed to express the PIASγ fragment in the correct reading frame comprising residue 7–144 from exon 1 and 2, followed by 19 additional residues specific to this transcript (Protein accession number NP_056981.2; transcript ENSMUST00000129549). Of note, the Matchmaker cDNA library was generated using an oligo(dT) reverse primer, presumably resulting in enrichment of this PIASγ cDNA which contains a polyA sequence in retained intron 2.

### HEK293T cell culture and immunoprecipitation

HEK293T cells were cultured in DMEM medium (Invitrogen) containing 10% fetal calf serum, 1% non-essential amino acids (Gibco) and 1% penicillin/streptomycin (Gibco). They were seeded one day before transfection and transfected at a confluency of 20%. Medium was refreshed 1 h before transfection, which was done with DNA precipitates formed in 125 mM CaCl_2_ in HEBS (140 mM NaCl, 25 mM HEPES, 0.75 mM Na_2_HPO_4_, pH 7.05). Medium was replaced 12 h after transfection and cells were harvested in 800 µl lysis buffer on ice (50 mM Tris pH 7.5, 1% Triton X-100, 1.5 mM MgCl_2_, 5 mM EDTA, 100 mM NaCl, protease inhibitor) when 100% cell confluency was reached. 20 mM N-ethylmaleimide was added to the lysis buffer in the indicated experiments. After centrifugation at 4°C, 2.5% of the lysate was kept aside to use as a control for protein input. The rest of the lysate was precleared with 50 µl PBS washed 10% Protein A agarose beads (Sigma) for 1 h at 4°C. Meanwhile, 50 µl 10% Protein A agarose beads were preblocked with 50 mM Tris pH 7.5, 1% Triton X-100, 1.5 mM MgCl_2_, 5 mM EDTA, 100 mM NaCl and 20% glycerol. Precleared lysate was then incubated with blocked beads and 0.5 µl antibody (mouse anti-FLAG (Sigma) or mouse anti-myc (Roche)) per immunoprecipitation for at least 2 h at 4°C. After washing with 5 subsequent low (100 mM NaCl) and high (500 mM NaCl) salt washes, beads and input control sample were analyzed by Western blotting.

### Western blot

Samples were boiled for 5 minutes at 100°C in Laemmli sample buffer and loaded onto a 6% SDS-PAGE gel. After wet protein transfer to a PVDF membrane for 2 h at 350 mA at 4°C, aspecific antibody binding to the membrane was prevented by incubation of the membrane with blocking solution (5% w/v milk powder and 0.2% Tween-20 in Tris-buffered saline (TBS) for 1 h at 4°C. Primary antibody incubation was done for 16 h at 4°C. After washing with TBS the membrane was stained with secondary antibody conjugated to alkaline phosphatase (AP; DAKO, 1∶5000 in TBS) for 1 h at 4°C. After washing again, the AP conjugate was visualized using ECF substrate (GE Healthcare). The membrane was scanned with a Fujifilm FLA-5000 Reader. Primary antibodies used were 1∶1000 rabbit anti-tomosyn-1/2 (kind gift from Dirk Fasshauer; [34]), 1∶2000 mouse anti-FLAG (Sigma), 1∶500 rabbit anti-SUMO-1 (Abcam) and 1∶1000 rabbit anti-SUMO-2/3 (Abcam).

### SUMOplot prediction and protein alignment

SUMOylation sites were predicted in the medium isoforms of mouse tomosyn-1 and tomosyn-2 using SUMOplot (http://www.abgent.com/SUMOplot). Alignments were done using Clustal Omega (http://www.ebi.ac.uk/Tools/msa/clustalo/). Protein accession numbers are: NP_001121187.1 (Homo sapiens tomosyn-1), NP_055795.1 (homo sapiens tomosyn-2), NP_001074813.2 (mouse tomosyn-1), AAT68175.1 (mouse tomosyn-2), NP_110470.1 (rat tomosyn-1), AAX89145.1 (C. elegans tomosyn), NP_727629.2 (Drosophila tomosyn), NP_004515.2 (Homo sapiens Lgl), NP_001152877.1 (mouse Lgl1), NP_001239461.1 (mouse Lgl2), NP_690057.1 (rat Lgl), CCD70868.1 (C. elegans Lgl), AAG22255.1 (Drosophila Lgl), NP_015357.1 (yeast Sro7), NP_009444.1 (yeast Sro77).

## Results

### Tomosyn-1 interacts with the SUMO E3 ligase PIASγ

**Figure 1:**
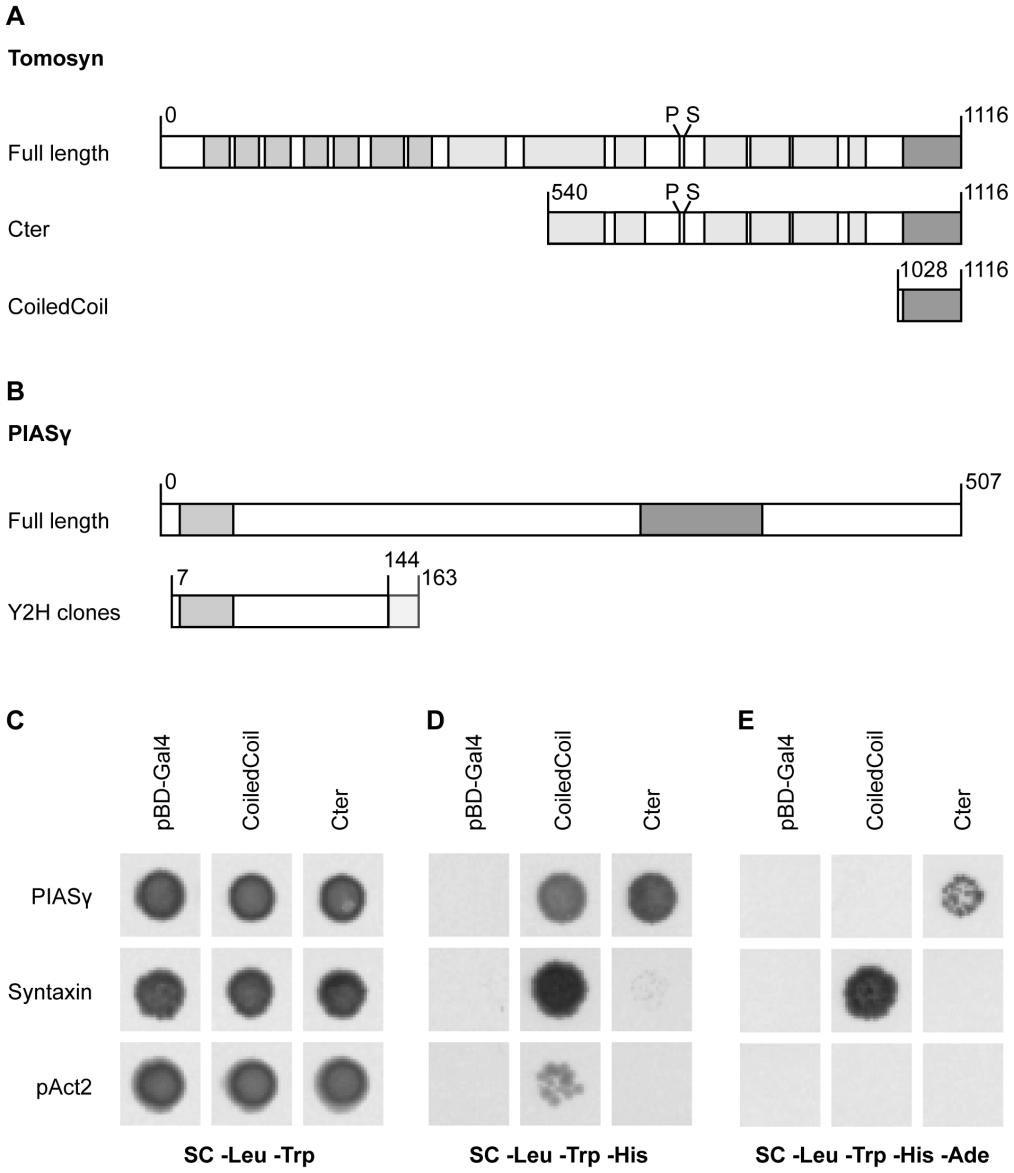
Yeast two-hybrid interaction of tomosyn-1 and PIASγ. (A) Tomosyn amino acids 540-1116 (Cter) or 1028-1116 (CoiledCoil) were used as bait in a yeast two-hybrid (Y2H) experiment (light grey: β-propeller domains, dark grey: synaptobrevin-like coiled coil domain, phosphorylation (P) and SUMOylation (S) sites are also depicted), next to an empty bait vector pBD-GAL4, in combination with prey constructs expressing (B) PIASγ (light grey: DNA-binding SAP-domain, dark grey: MIZ-type zinc finger domain) or syntaxin sequences, next to an empty prey vector pAct2. Yeast colonies were incubated on (C) histidine containing medium (no selection), or medium (D) lacking histidine (medium stringent selection) or (E) lacking histidine and adenine (stringent selection). Stringent selection indicated a strong PIASγ interaction with the larger Cter fragment of tomosyn-1 as well as syntaxin binding to tomosyn-1 CoiledCoil fragment.

In order to find novel protein interactors, C-terminal fragments of tomosyn-1 were used as bait in a yeast two-hybrid experiment. The long fragment (Cter) comprised amino acids 540–1116, while the short fragment (CoiledCoil) contained mainly the C-terminal coiled coil domain (amino acids 1028–1116) (Figure 1A). As controls, empty bait (pBD-GAL4) and prey (pAct2) vectors were used. Upon interaction of the bait and prey proteins, GAL4 transcription leads to expression of the HIS3 and ADE2 selectable marker genes [46]. We used a prey vector encoding syntaxin-1 [33] as a positive control. Syntaxin-expressing clones survived stringent selection (i.e. in absence of both histidine and adenine) only in combination with the CoiledCoil fragment (Figure 1). In contrast, syntaxin did not confer growth capacity to the Cter construct even under less stringent conditions (in absence of histidine, but presence of adenine). The limited GAL4 activation in Cter-expressing cells may reflect a reduced affinity of the bait and prey proteins or, alternatively, could be caused by steric hindrance.

Using a mouse brain cDNA library in combination with the tomosyn Cter bait construct, six independent clones with an in-frame cDNA insertion were identified that encoded the same fragment of PIASγ. The fragments comprised PIASγ amino acids 7–144, encoded by exon 1 and 2, plus 19 residues specific to this transcript (Figure 1B; transcript accession number ENSMUST00000129549). PIASγ is a 507 amino acid long E3 SUMO-protein ligase, also known as PIAS4 (protein inhibitor of Stat 4) or ZMIZ6 (Zn finger, MIZ-type containing 6). Despite the presence of a DNA-binding SAP domain in the PIASγ fragment, the prey construct alone did not confer growth capacity to the yeast cells, thus excluding a false-positive hit by auto-activation. Although the short tomosyn-1 CoiledCoil fragment was sufficient for PIASγ-dependent growth at medium stringency, the longest bait fragment Cter tolerated the highest selection stringency (Figure 1). In conclusion, the C-terminal domain of tomosyn-1 interacts with an N-terminal region of PIASγ, suggesting that its E3 SUMO ligase activity may be responsible for the SUMOylation of tomosyn-1[40].

### PIASγ / tomosyn-1 interaction is NEM dependent

**Figure 2:**
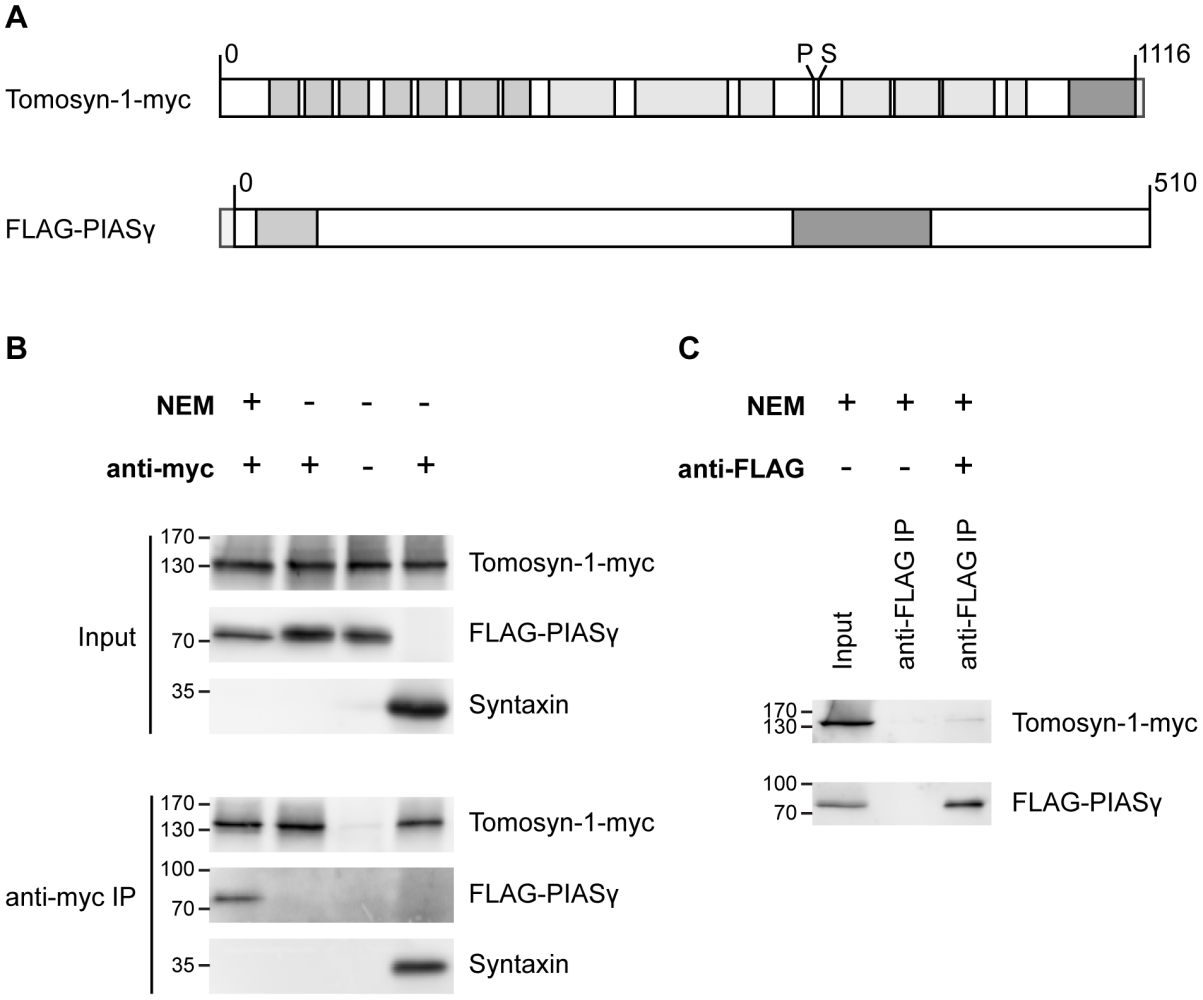
NEM dependent PIASγ / tomosyn-1 interaction. (A) Schematic representation of the constructs used in immunoprecipitation experiments. Tomosyn light grey: β-propeller domains, tomosyn dark grey: synaptobrevin-like coiled coil domain. Tomosyn phosphorylation (P) and SUMOylation (S) sites are also depicted. A small myc-tag is depicted at the tomosyn C-terminus. PIASγ light grey: DNA-binding SAP-domain, dark grey: MIZ-type zinc finger domain. The N-terminal FLAG-tag is also depicted. (B) FLAG-PIASγ co-precipitates with full-length tomosyn-1-myc in the presence of N-ethylmaleimide (NEM). Lysate from HEK293T cells co-transfected with tomosyn-1-myc (126 kDa) and FLAG-PIASγ (57 kDa, but reported to run higher on a Western blot ([56]) was subjected to immunoprecipitation using an anti-myc antibody with/without NEM in the lysis buffer. Antibody was omitted in the negative control. In the positive control, syntaxin (35 kDa) was expressed instead of FLAG-PIASγ. A fraction (2.5%) of the total cell lysate was loaded on the gel to verify protein expression (‘input’), the rest of the sample was used for immunoprecipitation (‘anti-myc IP’). (C) Tomosyn-1-myc co-precipitated in a reverse immunoprecipitation with an anti-FLAG antibody pulling down FLAG-PIASγ in the presence of NEM.

To validate the above results, we performed immunoprecipitation experiments using tagged full-length versions of tomosyn-m1 and PIASγ expressed in HEK293T cells (Figure 2A). Consistent with the yeast two-hybrid results, FLAG-PIASγ co-precipitated with tomosyn-m1-myc, whereas it did not co-precipitate in a negative control sample where the anti-myc antibody was omitted. Syntaxin was again used as a positive control. Notably, the interaction was only detected in the presence of N-ethylmaleimide (NEM; Figure 2B). SUMOylation is known to be rapidly reversible and often the SUMO-modified form is a minor species [48]. NEM stabilizes SUMO conjugates by covalent modification of the sulfhydryl group of the catalytic cysteine residue on SUMO-specific proteases (SENPs). Thus, SUMOylation of tomosyn and/or PIASγ could be required for co-immunoprecipitation. In a reversed immunoprecipitation experiment, NEM was again included and an anti-FLAG antibody was used to pull down PIASγ. This suggested a weak interaction of PIASγ with tomosyn (Figure 2C). In conclusion, these complementary experiments validate the PIASγ – tomosyn interaction observed in the yeast two-hybrid screen and suggest that it is SUMOylation-dependent.

### Tomosyn-1 is preferentially modified by SUMO-2/3

**Figure 3:**
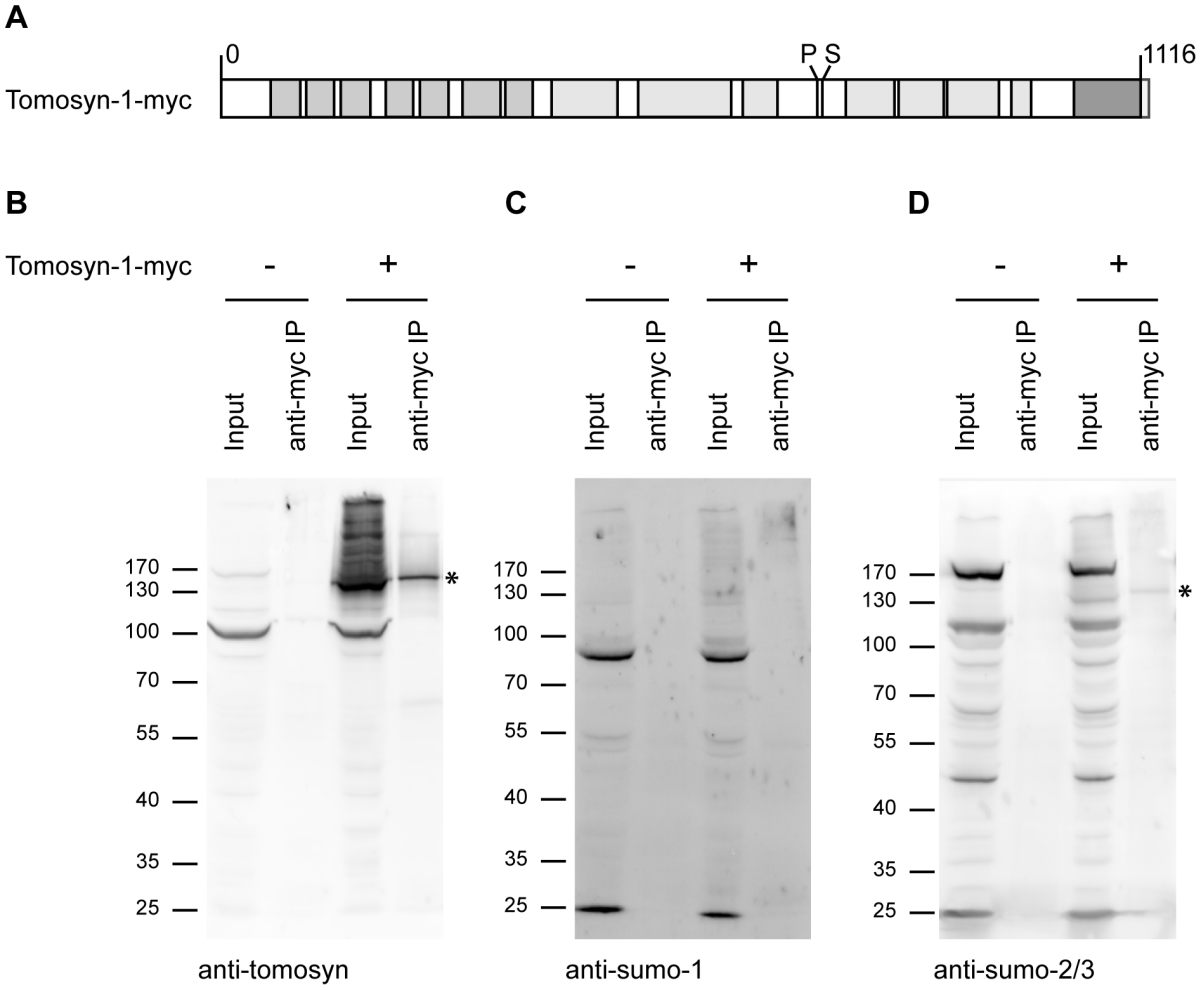
SUMO-2/3 modification of tomosyn-1. (A) Schematic representation of the tomosyn-m1 construct used in the immunoprecipitation experiment. Light grey: β-propeller domains, tomosyn dark grey: synaptobrevin-like coiled coil domain. Tomosyn phosphorylation (P) and SUMOylation (S) sites are also depicted. A small myc-tag is depicted at the tomosyn C-terminus. Immunoprecipitation of mock- and tomosyn-1-myc transfected HEK293T cell lysates with an anti-myc antibody was analyzed by immunoblotting with (B) anti-tomosyn, (C) anti-SUMO-1 or (D) anti-SUMO-2/3 antibodies. A fraction (2.5%) of the total cell lysate was loaded on the gel to verify protein expression (‘input’), the rest of the sample was used for immunoprecipitation (‘anti-myc IP’). The SUMO-2/3 antibody detected a band the size of tomosyn-1-myc (*; 126 kDa), indicating SUMOylation of tomosyn by SUMO-2/3.

To test if tomosyn-1 is subject to SUMOylation by SUMO-1 in addition to SUMO-2/3 [40], HEK293T cells were transfected with myc-tagged tomosyn-m1 (Figure 3A). SUMO-1, SUMO-2/3 and PIASγ are natively expressed in HEK293T cells [49], [50], [51]. Tomosyn pulldown by anti-myc immunoprecipitation was confirmed with an antibody against tomosyn (Figure 3B). Staining with an anti-SUMO-1 antibody (Figure 3C) did show immunoreactivity in the input (the prominent <100 kDa band likely reflecting SUMOylated RanGAP1; molecular mass ∼90 kDa; [5], [52], but not in the immunoprecipitated sample. On the other hand, probing the blot with a SUMO-2/3 antibody (Figure 3D) resulted in a specific band at the same height as tomosyn-m1-myc after immunoprecipitation (Figure 3B, tomosyn band is highlighted with an asterisk). In the negative control experiment, using mock transfected cells, this band was not observed. This suggests that tomosyn-1 is SUMOylated by SUMO-2/3 specifically.

## Discussion

Yeast two-hybrid screening revealed PIASγ as a novel interaction partner of the tomosyn-1 C-terminus. The interaction was confirmed by reciprocal co- immunoprecipitation from transfected HEK293T cells. Specific SUMO-2/3 attachment to tomosyn-1 was observed. Consistent with this finding, a binary interaction between SUMO-2/3 and PIASγ has been reported [53].

### Several tomosyn SUMOylation sites are predicted using a consensus sequence

**Figure 4:**
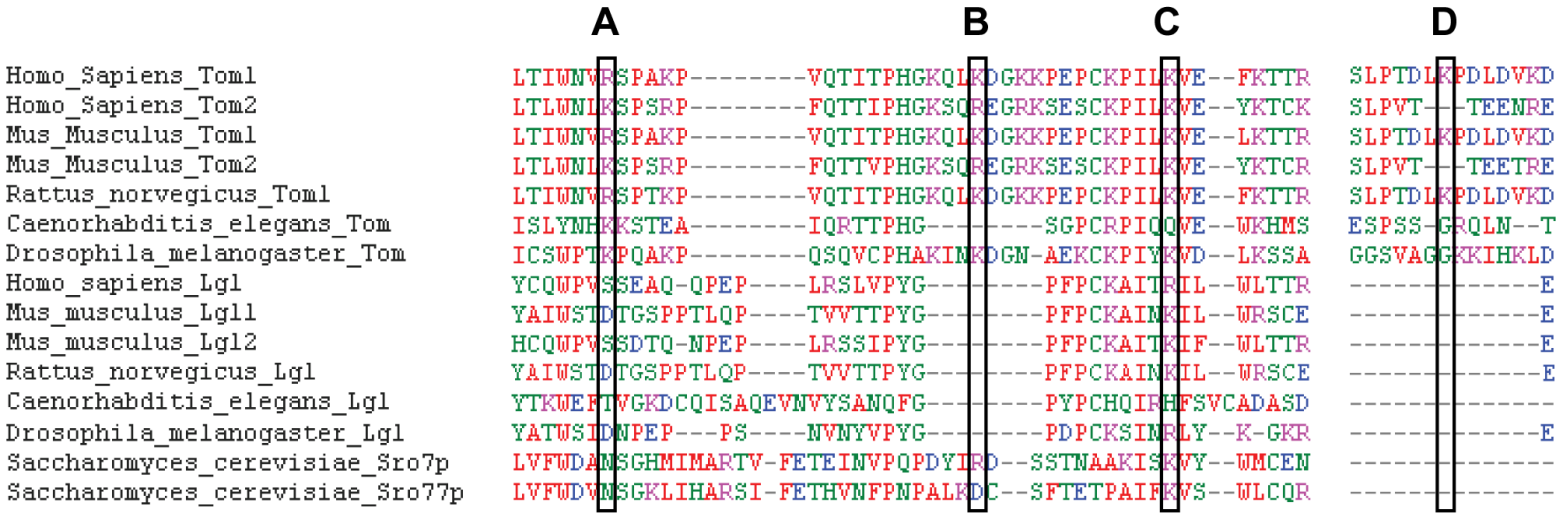
Conservation of SUMOplot predicted tomosyn SUMOylation sites. Protein alignment of tomosyn isoforms in different species, as well as the homologous proteins Lgl and yeast Sro7 and Sro77. Lysine residues that are predicted to be subject to SUMOylation in mouse tomosyn are highlighted with black boxes. SUMOplot prediction scores for (A) tomosyn-m2 K279, (B) tomosyn-m1 K285, (C) tomosyn-m1 K298 / tomosyn-m2 K309 and (D) tomosyn m-1 K730 are shown in table 1. Colours represent amino acid physicochemical properties: small (red), acidic (blue), basic (magenta), hydroxyl/sulfhydryl/amine/G (green).

SUMO substrates can be recognized by a ψ-K-x-D/E motif, in which ψ is a hydrophobic residue, K is the SUMO attaching lysine, x is any amino acid and D or E is an acidic residue [54], [55]. Some SUMOylated proteins lack this kind of consensus sequence however. Possible SUMOylation sites on mouse tomosyn-1 were previously predicted using SUMOplot [40], which uses this consensus sequence to calculate the probability of SUMO ligation. One of the three most likely sites is tomosyn-m1 lysine K730 (Table 1) and mutation of this residue to an arginine (R) causes a loss of SUMOylation [40]. K730 is specific to splice variants tomosyn-m1 and -b1 and absent in tomosyn-s1 due to exon skipping. The site is also absent in tomosyn orthologs from *C. elegans* or *D. melanogaster*, and in various paralogs (tomosyn-2, lethal giant larvae homologs Mgl/Lgl and the yeast Sro7 and Sro77 homologs; Figure 4D). Therefore, SUMOylation of tomosyn-1 at this residue is not likely required for its basal function, but might fine-tune the function of tomosyn-1 in mammals.

**Table 1:** SUMOplot SUMOylation prediction sites on tomosyn-m1 [40] and tomosyn-m2.

	Amino acid number	Sequence	SUMOplot score
**Tomosyn-m1**	K298 (Figure 4C)	PCKPI**LKVE**LKTTR	0.91
	K730 (Figure 4D)	SLPTD**LKPD**LDVKD	0.91
	K285 (Figure 4B)	PHGKQ**LKDG**KKPEP	0.73
**Tomosyn-m2**	K309 (Figure 4C)	SCKPI**LKVE**YKTCR	0.91
	K279 (Figure 4A)	LTLWN**LKSP**SRPFQ	0.8

The SUMO c onsensus sites are depicted in bold, while the actual lysine predicted to be subject to SUMOylation is underlined. The reported sites also exist in the other splice variants, except for the K730 site that is absent in tomosyn-s1.

### Full-length immunoprecipitation of PIASγ and tomosyn is NEM dependent

NEM-mediated stabilization of SUMO conjugation was required to detect the interaction between the full-length proteins in immunoprecipitation experiments, suggesting that SUMOylation is required for their interaction. Since the cellular machinery for SUMO modification is conserved in yeast, it is possible that the interaction in the yeast two-hybrid system also involved SUMOylated proteins. All prey clones contained the lysine K35 target site for PIASγ SUMOylation [56]. From the bait constructs, only the longer Cter tomosyn-1 fragment contained the K730 SUMOylation site. The weaker interaction with the shorter CoiledCoil construct suggests that this region contributes partially to the PIASγ interaction. Thus, SUMOylation of both tomosyn and PIASγ could contribute to their interaction. However, alternative NEM-induced mechanisms may also be involved. For example, a contribution of N-ethylmaleimide sensitive factor (NSF) inactivation and thereby accumulation of HEK293T endogenous SNARE complexes [57], cannot be excluded.

### Possible role of tomosyn SUMOylation in synaptic plasticity

A number of neuronal and synaptic processes are regulated by SUMOylation, including synapse formation and the regulation of neuronal activity [17]. Post-synaptically, endocytosis of kainate receptor subunits is regulated by SUMOylation [42], [58], [59]. Furthermore, glutamatergic neurotransmission is influenced by presynaptic protein SUMOylation [60], for example via SUMOylation of group III metabotropic glutamate (mGlu) receptors [59]. In view of tomosyn's role in synaptic glutamate release, tomosyn SUMOylation likely represents a mechanism to control synaptic plasticity. The reversible character of SUMO ligation is ideally suited to adapt synaptic strength to the dynamic environment. The fact that SUMOylation levels in neurons can be modulated in an activity-dependent manner strengthens this idea [10], [60], [61]. Tomosyn-mediated inhibition of growth hormone secretion from high potassium depolarized PC12 cells is more prominent upon mutation of the tomosyn-m1 K730 SUMOylation site, thus confirming that tomosyn SUMOylation regulates the strength of secretory inhibition. This is independent of its interaction with syntaxin, which is unaltered in a FRET (**Fluorescence Resonance Energy Transfer**) assay with SUMOylation deficient tomosyn [40]. Inhibitory activity is regulated by intramolecular interactions of tomosyn [29], [62] that might require SUMOylation to induce a conformational switch. Tomosyn SUMOylation could contribute to synaptic plasticity via such regulation of inhibitory strength.

### Potential interplay between tomosyn phosphorylation and SUMOylation

Phosphorylation of a protein can affect its SUMOylation state negatively [44] or positively [43]. In neurons, SUMOylation of the kainate receptor subunit GluK2 depends on phosphorylation by Protein kinase C [42]. A functional tomosyn-1 PKA phosphorylation site at amino acid position 724 has been reported, which is only 6 amino acids upstream of the K730 SUMOylation site (Figure 1A). Interestingly, cAMP-dependent PKA phosphorylation of tomosyn-1 facilitates neurotransmitter release by reducing tomosyns inhibitory interaction with syntaxin [41]. SUMOylation could contribute to the effect of PKA phosphorylation of tomosyn-1 on synaptic plasticity. This is supported by the suggestion that increased protein SUMOylation is required for long term potentiation (LTP; [9]). Also ischemic stress induces LTP [63] and enhances SUMO-2/3 modification specifically [11], [12], [13], [14], [15]. Modification of tomosyn by phosphorylation and SUMOylation may thus co-regulate its function.

Taken together, we conclude that the PIASγ-mediated SUMOylation of tomosyn is likely to contribute to overall synaptic plasticity. This pathway may be regulated by PKA-phosphorylation and ischemic stress. The implications of this novel mechanism in synaptic regulation remain to be assessed.
